# Genome Sequence of *Pectobacterium atrosepticum* Strain 21A

**DOI:** 10.1128/genomeA.00935-14

**Published:** 2014-09-18

**Authors:** Yevgeny Nikolaichik, Vladimir Gorshkov, Yuri Gogolev, Leonid Valentovich, Anatoli Evtushenkov

**Affiliations:** a Department of Molecular Biology, Faculty of Biology, Belarus State University, Minsk, Belarus; b Kazan Institute of Biochemistry and Biophysics, Kazan Scientific Center, Russian Academy of Sciences, Kazan, Russia; c Institute of Microbiology, National Academy of Sciences, Minsk, Belarus

## Abstract

We report the annotated genome sequence of the enterobacterial plant pathogen *Pectobacterium atrosepticum* strain 21A, isolated in Belarus from potato stem with blackleg symptoms.

## GENOME ANNOUNCEMENT

*Pectobacterium* spp. are capable of causing disease in a wide spectrum of plant species. However, *Pectobacterium atrosepticum* is characterized by a rather narrow host range and is mostly associated with blackleg and soft rot diseases in potato. The *P. atrosepticum* strain 21A was isolated in Belarus in 1978 (1) from potato stem with blackleg symptoms. The strain is virulent in potato and differs from the well-characterized *P. atrosepticum* strain SCRI 1043 (2) in its ability to cause hypersensitive reactions in nonhost plants.

The data for the genome assembly were generated using Illumina MiSeq and the Nextera XT library preparation protocol. After quality filtering with Prinseq (http://prinseq.sourceforge.net), 12,238,721 reads were retained, of which 98% mapped to the final assembly, giving a coverage of 268. Filtered reads were assembled into 35 large (>1,000 bp) contigs using SPAdes (3) with BayesHammer (4) error correction. SSPACE (5) and GapFiller (6) were used for initial gap closure, followed by manual resolution of repeats, with the genome sequence of *P. atrosepticum* strain SCRI 1043 used to assist in scaffolding.

The complete genome of *P. atrosepticum* strain 21A consists of a 4,991,806-bp chromosome with a GC content of 51.1% and a 32,444-bp plasmid with a GC content of 47%. Based on the difference in coverage of the two replicons, the plasmid is present in 3 to 4 copies per cell.

Genome annotation was performed using the Prokka annotation pipeline (7). Coding sequences were predicted using Prodigal (8), signal peptides by SignalP (9). tRNA genes and transfer-messenger RNA (tmRNA) were predicted by ARAGORN (10), rRNA genes by Barrnap (http://www.vicbioinformatics.com/software.barrnap.shtml), and noncoding RNAs- by Infernal (11). Clustered regularly interspaced short palindromic repeats (CRISPRs) were detected by MinCED (https://github.com/ctSkennerton/minced). The genome contains 4,424 protein coding sequences and 22 rRNA genes organized into 7 operons, 77 tRNAs, and 2 CRISPR loci. The genome codes for a set of extracellular hydrolases typical for pectolytic bacteria, including 9 pectate lyases, 4 polygalacturonases, 1 cellulase, 2 hemicellulases, and an extracellular protease. All six known types of protein secretion systems are present.

Organization of the *P. atrosepticum* 21A chromosome is very similar to that of three other known *P. atrosepticum* genomes. Overall, gene content and order are the same in the four strains, with the exception of horizontally transferred sequences (mostly phage related), which account for <100 genes unique for *P. atrosepticum* 21A. Another notable difference between *P. atrosepticum* chromosomes is a large (1.35- Mb) inversion in *P. atrosepticum* strains 21A and CFBP6276 (12) relative to SCRI 1043 and the recently sequenced genome of strain JG10-08 (GenBank accession no. CP007744).

The plasmid in *P. atrosepticum* 21A has weak similarity to the plasmid-like sequence integrated into the SCRI 1043 chromosome. The similarity, however, is restricted to the type IV secretion genes that might be responsible for conjugative transfer and the antirestriction gene. Compared to the plasmid-like element in SCRI 1043, the *P. atrosepticum* 21A plasmid lacks arsenical resistance genes but has genes that might be related to pathogenicity, including genes coding for a phospholipase and an H-NS-like protein.

### Nucleotide sequence accession numbers.

The nucleotide sequence accession numbers are CP009125 and CP009126 for the chromosome and the plasmid, respectively.
